# Associations Between Anemia and Obstructive Sleep Apnea in Patients with Cystic and Non-Cystic Bronchiectasis: A Prospective Cross-Sectional Study

**DOI:** 10.3390/biomedicines14040915

**Published:** 2026-04-17

**Authors:** Baran Balcan, Duygu Vezir, Ramazan Ocal, Caner Cinar, Erdal Aksoy, Yeliz Celik, Berrin Ceyhan

**Affiliations:** 1 Department of Pulmonary Medicine, Koç University School of Medicine, 34450 Istanbul, Türkiye; 2 Department of Pulmonary Medicine and Intensive Care, Sureyyapasa Teaching and Education Hospital, 34890 Istanbul, Türkiye; 3 Department of Pulmonary Medicine and Intensive Care, Faculty of Medicine, Marmara University, 34890 Istanbul, Türkiye; 4 Department of Hematology, LIV Hospital, 06690 Ankara, Türkiye; 5 Department of Administration and Forensic Medicine, Koç University Hospital, 34450 Istanbul, Türkiye; 6 Koc University Research Center for Translational Medicine, Koc University, 34450 Istanbul, Türkiye

**Keywords:** anemia, bronchiectasis, obstructive sleep apnea, cystic fibrosis (CF), clinical outcomes

## Abstract

**Background**: Anemia and obstructive sleep apnea (OSA) are prevalent comorbidities in bronchiectasis and may share overlapping pathophysiological mechanisms. However, their combined impact on clinical outcomes in bronchiectasis remains underexplored. We aimed to investigate the associations between anemia, OSA, and clinical characteristics in patients with bronchiectasis, including cystic fibrosis (CF) and non-CF subtypes. **Methods**: 70 adults with bronchiectasis (35 CF-related, 35 non-CF) underwent polysomnography. Anemia was defined using standard hemoglobin < 13 g/dL for men and <12 g/dL for women. Clinical, nutritional, and sleep-related variables were assessed, and associations with anemia were evaluated. **Results**: Anemia was present in 24.3% of participants. Compared to non-anemic patients, those with anemia had significantly higher rates of female sex (38.5% vs. 6.5%, *p* = 0.002), nutritional problems (47.1% vs. 20.8%, *p* = 0.034), OSA prevalence (94.1% vs. 54.7%, *p* = 0.003), and annual hospitalizations (1.41 ± 0.41 vs. 0.43 ± 0.10, *p* = 0.002). In multivariate analysis, female sex (OR: 12.32; 95% CI: 3.12–45.96; *p* = 0.002) and OSA (OR: 4.70; 95% CI: 2.67–45.29; *p* = 0.007) remained independent predictors of anemia. Subgroup analysis showed a significant univariate association between OSA and anemia in CF patients (*p* = 0.045), while hospitalization frequency was an independent predictor of anemia in non-CF bronchiectasis (*p* = 0.040). **Conclusions**: Anemia in bronchiectasis is independently associated with female sex and OSA, with additional exploratory subgroup findings. These findings indicate an association between OSA and anemia within bronchiectasis populations; however, the cross-sectional design precludes conclusions regarding directionality or causality.

## 1. Introduction

Obstructive sleep apnea (OSA) is a prevalent sleep disorder characterized by recurrent episodes of partial or complete upper airway obstruction during sleep, resulting in intermittent hypoxia, sympathetic activation, and sleep fragmentation [1,2]. In recent years, increasing attention has been directed toward the prevalence and clinical implications of OSA in patients with chronic pulmonary diseases, particularly bronchiectasis, a condition defined by permanent bronchial dilation, impaired mucociliary clearance, and chronic airway inflammation [3,4].

Both cystic fibrosis (CF)-related and non-CF bronchiectasis have been associated with an increased risk of sleep-disordered breathing (SDB) [5]. Structural lung damage, mucus plugging, and airflow limitation may compromise nocturnal ventilation and gas exchange, predisposing patients to more severe desaturation events during sleep [6,7]. Prior studies have demonstrated that the prevalence of OSA in these populations is often higher than in the general population [8,9].

In addition, the pathophysiological burden of OSA may be further amplified by comorbid systemic conditions such as anemia, which is frequently observed in bronchiectasis due to chronic inflammation, recurrent infections, and nutritional deficiencies. In CF patients, malabsorption syndromes may further contribute to anemia development [10,11]. Anemia reduces the oxygen-carrying capacity of blood and may therefore exacerbate the adverse effects of OSA-related intermittent hypoxia [10,12,13].

Despite this plausible pathophysiological interplay, the relationship between anemia and OSA in bronchiectasis remains insufficiently explored. Most previous studies have evaluated these conditions independently, addressing their potential combined impact on sleep-related oxygenation and clinical outcomes. A better understanding of this may improve risk stratification and identify potential therapeutic targets in this vulnerable patient population. Therefore, the present study aimed to investigate the associations between anemia and OSA in patients with bronchiectasis and to explore whether anemia is associated with differences in sleep-related parameters. Given the cross-sectional design, the objective was not to establish causality but rather to identify clinically relevant associations.

## 2. Materials and Methods

### 2.1. Study Design and Participants

**Figure 1:**
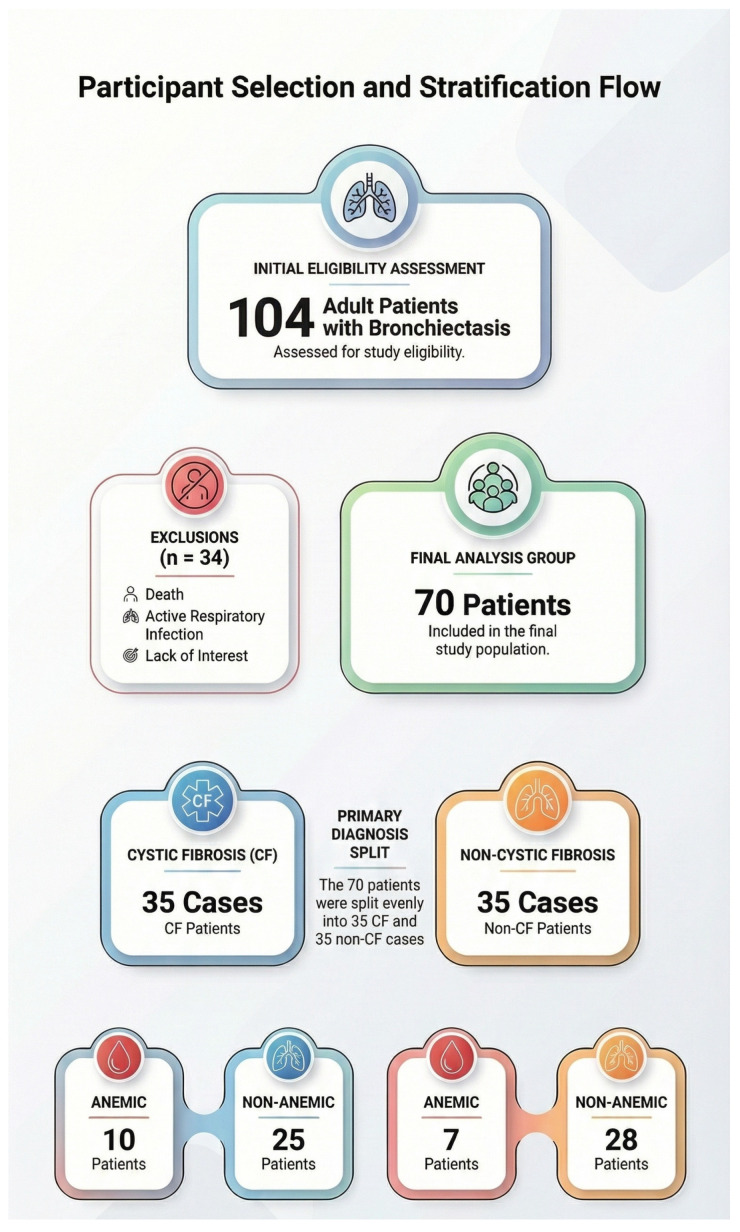
Flow chart of the participants.

This prospective, cross-sectional study was conducted between April 2019 and December 2021. Adult patients (≥18 years) diagnosed with bronchiectasis, including both cystic fibrosis (CF)-related and non-CF bronchiectasis, were recruited from the Adult Chest Diseases Outpatient Clinic at Marmara University Pendik Training and Research Hospital (Figure 1). A total of 104 patients were initially assessed for eligibility. Of these, 34 were excluded due to refusal to participate in further evaluation, death during follow-up, or the presence of active respiratory infection at the time of assessment. The final study consisted of 70 participants, equally distributed between the CF (n = 35) and non-CF (n = 35) bronchiectasis groups.

Anemia was defined according to standard hemoglobin thresholds (<13 g/dL for men and <12 g/dL for women). The study received approval from the Marmara University Institutional Ethics Committee (protocol number: 09.2019.503) and was conducted in accordance with the Declaration of Helsinki. Written informed consent was obtained from all participants prior to study enrollment, in accordance with the Declaration of Helsinki.

### 2.2. Polysomnographic Evaluation

All subjects underwent overnight attended polysomnography (PSG) using the Embletta MPR device (Natus Medical Incorporated, Orlando, FL, USA). The PSG recordings included assessment of sleep architecture, total sleep duration, nasal airflow, thoracoabdominal movements, body posture, heart rate, oxygen saturation (SpO_2_), leg muscle activity, periodic limb movements, and snoring events. If total sleep time was less than 240 min, a repeat PSG was offered. All recordings were scored and interpreted according to the American Academy of Sleep Medicine (AASM) criteria by a single experienced physician (B.B) to ensure consistency across evaluations [14].

### 2.3. Participant Stratification

As shown in Figure 1, patients were categorized according to anemia status for eligibility. Only participants who completed an in-laboratory full-night PSG with a total sleep time of at least 4 h were included in the final analysis.

### 2.4. Assessment Tools

#### 2.4.1. Epworth Sleepiness Scale (ESS)

Daytime sleepiness was assessed using the validated Turkish version of the Epworth Sleepiness Scale (ESS), a self-report questionnaire consisting of eight items scored from 0 to 3. The total score ranges from 0 to 24, with scores ≥11 indicating excessive daytime sleepiness (EDS) [15].

#### 2.4.2. Charlson Comorbidity Index

The Charlson Comorbidity Index was used to quantify comorbidity burden and estimate 1-year mortality risk. Higher scores indicate a greater burden of comorbid conditions [16].

#### 2.4.3. Modified Medical Research Council (mMRC) Dyspnea Scale

Dyspnea severity was assessed using the mMRC scale, a five-point scale ranging from 0 (no dyspnea) to 4 (severe dyspnea with minimal exertion) [17].

#### 2.4.4. Zung Self-Rating Depression Scale (SDS)

Depressive symptoms were evaluated using the Turkish version of the Zung SDS [18]. This self-administered instrument includes 20 items, each scored on a 4-point scale from 1 (“a little of the time”) to 4 (“most of the time”). Reverse scoring was applied to items 2, 5, 6, 11, 12, 14, 16, 17, 18, and 20. A total score of ≥40 was considered indicative of depressive symptoms. All responses were entered into the study database by a designated investigator.

### 2.5. Statistical Analysis

Statistical analyses were performed using IBM SPSS Statistics for Windows, Version 22.0 (IBM Corp., Armonk, NY, USA). Continuous variables were summarized as mean ± standard deviation (SD) if normally distributed or as median with interquartile range (IQR) if not. Categorical data were reported as counts and percentages. Comparisons between groups were conducted using the *t*-test for normally distributed continuous variables, the Mann–Whitney U test for non-normally distributed data, and the chi-square test (or Fisher’s exact test when appropriate) for categorical variables. Logistic regression models were employed to examine associations between sleep apnea and clinical variables, with results reported as odds ratios (ORs) with 95% confidence intervals (CIs). All tests were two-tailed, and statistical significance was defined as a *p*-value < 0.05.

## 3. Results

### 3.1. Demographics, Clinical, and Sleep-Related Characteristics

As shown in Table 1, a total of 70 participants were included in the analysis, of whom 17 were classified as having anemia and 53 as non-anemic. The proportion of female participants was significantly higher in the anemia group compared to the non-anemia group (38.5% vs. 6.5%; *p* = 0.002). Nutritional support was also more frequently observed among the patients with anemia (47.1% vs. 20.8%; *p* = 0.034). The prevalence of OSA was significantly greater in the anemia group (94.1% vs. 54.7%; *p* = 0.003). In addition, patients with anemia had a significantly higher annual hospitalization rate (1.41 ± 0.41 vs. 0.43 ± 0.10; *p* = 0.002). No significant differences were observed between groups with respect to age, BMI, or smoking status. Similarly, comorbid conditions, including diabetes mellitus, pancreatic disease, cardiac disease, and the use of oxygen therapy, were comparable between groups. Measures of daytime sleepiness also did not differ significantly. Although the mean ESS score was higher in the anemia group, this difference did not reach statistical significance, and subjective sleepiness was also comparable. Likewise, no significant differences were observed in dyspnea severity (mMRC score), Charlson Comorbidity Index, disease duration, or annual exacerbation frequency. Polysomnographic parameters, including total sleep time, sleep efficiency, sleep latency, REM latency, AHI, ODI, mean oxygen saturation, lowest oxygen saturation, and time spent with SpO_2_ < 90%, were similar between groups. Although the average heart rate tended to be higher in the anemia group, this did not reach statistical significance.

### 3.2. Factors Associated with Anemia in the Overall Bronchiectasis Cohort

As presented in Table 2, in the univariate analysis, female sex was strongly associated with anemia (OR: 9.06; 95% CI: 1.88–43.62; *p* = 0.006). Hospitalization frequency was significantly associated with anemia (OR:1.99; 95% CI: 1.21–3.23; *p* = 0.006). In addition, nutritional support (OR: 3.39; 95% CI: 1.06–10.83; *p* = 0.039) and the presence of OSA (OR: 3.42; 95% CI: 1.64–7.21; *p* = 0.015) were significantly associated with anemia. Other variables, including age, BMI, smoking status, cystic fibrosis status, disease duration, mMRC score, Charlson Comorbidity Index, diabetes mellitus, pancreatic disease, and oxygen therapy use, were not significantly associated with anemia. Although ESS score and heart rate showed trends toward association (ESS: OR: 1.14; *p* = 0.080; heart rate: OR: 1.05; *p* = 0.095), these did not reach statistical significance. Additionally, no significant associations were found for polysomnographic parameters, including total sleep time, sleep efficiency, sleep latency, AHI, ODI, average or lowest oxygen saturation, or duration of desaturation below 90%. In the multivariate analysis, female sex remained an independent predictor of anemia (OR: 12.32; 95% CI: 3.12–45.96; *p* = 0.002). Similarly, OSA was also significantly associated with anemia (OR: 4.70; 95% CI: 2.67–45.29; *p* = 0.007). In contrast, hospitalization frequency and nutritional support were no longer significant after adjustment.

### 3.3. Factors Associated with Anemia in Patients with Cystic Fibrosis Bronchiectasis

As demonstrated in Table 3, in the cystic fibrosis subgroup, OSA was significantly associated with anemia in the univariate analysis (OR: 4.24; 95% CI: 1.88–12.25; *p* = 0.045). Female sex demonstrated a trend but did not reach statistical significance. Hospitalization frequency (OR: 1.68; *p* = 0.093) and Zung SDS score for depressive symptoms (OR: 1.06; *p* = 0.094) also showed borderline associations with anemia, although these did not achieve statistical significance. Other clinical variables, including age, BMI, smoking status, disease duration, MMRC score, Charlson index, diabetes, and nutritional support, were not significantly associated with anemia. Likewise, subjective sleepiness and the ESS score itself did not reach statistical significance. Polysomnographic parameters were not associated with anemia in this subgroup. In the multivariate analyses, no variables remained statistically significant.

### 3.4. Factors Associated with Anemia in Non-Cystic Fibrosis Bronchiectasis

As shown in Table 4, both hospitalization frequency (OR: 4.50; 95% CI: 2.47–42.5; *p* = 0.049) and OSA (OR: 2.85; 95% CI: 1.01–8.01; *p* = 0.046) were significantly associated with anemia in univariate analyses. Other variables, including age, female gender, BMI, smoking status, disease duration, mMRC score, Charlson Comorbidity Index, diabetes mellitus, and sleep-related measures, were not significantly associated with anemia. Nutritional support showed a trend toward association but did not reach statistical significance. In the multivariate model, hospitalization frequency remained an independent predictor of anemia (OR: 3.69; 95% CI: 1.06–12.84; *p* = 0.040), whereas OSA did not retain significance (OR: 1.12; 95% CI: 0.96–1.31; *p* = 0.136).

## 4. Discussion

In this prospective cross-sectional study of adults with bronchiectasis, we identified a clinically relevant association between anemia and OSA, along with relationships involving hospitalization frequency and nutritional status. Notably, female sex and the presence of OSA emerged as independent factors associated with anemia in the overall cohort.

The higher proportion of female participants in the anemia group is consistent with well-established epidemiological data demonstrating a greater prevalence of anemia among women, largely attributable to factors such as menstrual blood loss, pregnancy, and iron deficiency [19,20]. This finding may also reflect broader biological and sociocultural determinants influencing nutritional status and healthcare access.

Nutritional support was more frequently observed among patients with anemia, supporting the established link between nutritional deficiencies and anemia, particularly deficiencies in iron, folate, and vitamin B12 [21]. In patients with bronchiectasis, chronic inflammation, recurrent infections, and, in CF, malabsorption syndromes may further contribute to the development of anemia.

One of the most notable findings of this study is the significantly higher prevalence of OSA among patients with anemia, as well as an independent association between OSA and anemia in multivariate analysis. This observation is consistent with previous studies reporting associations between OSA, systemic inflammation, and hematological parameters. However, given the cross-sectional design, these findings should not be interpreted as evidence of a causal or mechanistic relationship. The observed association may instead reflect shared underlying factors such as systemic inflammation, nutritional status, or overall disease severity. Recent reviews have also highlighted the broader cardiovascular implications of OSA, further supporting its systemic impact beyond respiratory outcomes [22]. Previously, Khan et al. demonstrated that treatment of OSA in elderly patients was associated with changes in hemoglobin and hematocrit levels, supporting a potential link between intermittent hypoxia and hematological parameters [23]. Similarly, elevated ferritin levels, reflecting both iron storage and inflammation, have been associated with OSA severity independent of body mass index, indicating a shared inflammatory pathway [24]. Furthermore, population-based analyses have suggested a complex, potentially bidirectional relationship between iron metabolism and OSA risk [25]. Recent evidence also indicates that anemia and OSA may interact synergistically in the development of comorbid conditions such as hypertension [26].

Despite these biologically plausible mechanisms, the present study was not designed to evaluate causal pathways. Therefore, the observed associations should be interpreted with caution and not as evidence of a direct mechanistic relationship [23,27].

Although hospitalization frequency and nutritional support were significantly associated with anemia in univariate analysis, these associations did not persist in the adjusted model. This attenuation may reflect confounding by OSA or sex, or it may indicate that hospitalization and malnutrition act as mediators or downstream consequences rather than independent predictors. Nonetheless, their initial significance highlights the broader systemic impact of anemia in chronic lung disease and warrants further investigation [28,29]. Another important consideration is the potential role of overall disease severity as a confounder. Patients with anemia in our cohort had higher rates of hospitalization and more frequent nutritional problems, suggesting a more severe clinical phenotype. It is therefore possible that both anemia and OSA reflect increased systemic disease burden rather than a direct association between the two conditions. The absence of detailed markers of bronchiectasis severity, such as lung function parameters or validated severity scores, further limits the ability to fully adjust for this potential confounding [30].

Interestingly, no significant associations were observed between anemia and polysomnographic severity indices, including AHI, ODI, and oxygen saturation parameters. This finding suggests that the presence of OSA, rather than its severity, may be more relevant in relation to anemia. An alternative explanation is that the association observed with OSA presence, but not with severity metrics, may reflect limited statistical power, dichotomization effects, or residual confounding. It is also possible that OSA acts as a marker of overall disease burden rather than a direct physiological driver of anemia. Therefore, the threshold effect hypothesis remains speculative and requires confirmation in larger studies [31].

In the CF participants, OSA was significantly associated with anemia, although this association did not persist after adjustment. This may be partly explained by the limited number of events and the resulting wide confidence intervals. This may be partly explained by the limited number of events and the resulting wide confidence intervals. Previous studies have reported a high prevalence of anemia and iron deficiency in CF populations, often related to malabsorption and nutritional deficiencies, which may further complicate the relationship between OSA and hematological parameters [32]. Therefore, these findings should be considered exploratory.

In contrast, in the non-CF bronchiectasis subgroup, both OSA and hospitalization frequency were associated with anemia in univariate analysis. However, only hospitalizations remained an independent predictor in the multivariate model. This finding suggests that, in non-CF bronchiectasis, anemia may be more closely linked to disease burden and healthcare utilization than to sleep-disordered breathing per se. Nevertheless, given the limited sample size and number of events, these subgroup findings should be interpreted with caution and require confirmation in larger cohorts [33,34].

Overall, the present findings support the existence of a clinically relevant association between anemia and OSA in patients with bronchiectasis while also highlighting potential differences between CF and non-CF populations. These results underscore the importance of a multidimensional clinical assessment that considers both respiratory and systemic factors in this patient group [11,24,25].

Several limitations should be acknowledged. First, the cross-sectional design of the study precludes any causal inference regarding the relationship between anemia and OSA. Longitudinal studies are needed to clarify temporal and directional associations. Second, the relatively small sample size, particularly the limited number of anemia events (n = 17), raises concerns regarding statistical power and model stability. Given the number of candidate variables explored, there is a potential risk of overfitting in the multivariable models. This may have contributed to the wide confidence intervals observed and limited the precision and generalizability of the estimated effect sizes. Therefore, the regression findings should be interpreted as exploratory rather than definitive. T. Third, the lack of etiological characterization of anemia represents a major limitation. Anemia in bronchiectasis is heterogeneous and may result from iron deficiency, chronic inflammation, malabsorption, or other systemic factors. However, key laboratory parameters such as ferritin, transferrin saturation, mean corpuscular volume, vitamin B12, folate, and inflammatory biomarkers were not systematically assessed. Without these data, it is not possible to distinguish between different anemia subtypes or to explore underlying mechanisms. Therefore, the observed associations should be interpreted in the context of overall disease burden rather than specific pathophysiological pathways. Additionally, gastrointestinal comorbidities—including potential occult bleeding or neoplastic conditions—were not systematically evaluated and may represent unmeasured confounders. Finally, as this was a single-center study conducted in a tertiary referral setting, generalizability to broader bronchiectasis populations may be limited.

## 5. Conclusions

This study demonstrates an association between anemia and OSA in patients with bronchiectasis. Female sex and OSA were independently associated with anemia in the overall cohort, while subgroup analyses suggested potential phenotype-specific patterns; however, these findings should be interpreted as exploratory given the limited sample size and cross-sectional design. Larger, longitudinal studies incorporating detailed hematologic profiling are required to clarify temporal and mechanistic links.

## Figures and Tables

**Table 1:** Clinical, demographic, and polysomnographic characteristics of bronchiectasis patients according to anemia status (n = 70).

Variable	Anemia (n = 17)	Non-Anemia (n = 53)	*p*-Value
Age (years)	29.65 ± 9.12	29.81 ± 9.12	0.960
Female sex (%)	38.5	6.5	0.002
BMI (kg/m^2^)	21.13 ± 4.16	22.91 ± 4.18	0.131
Current or former smoker (%)	5.9	15.1	0.323
Nutritional support (%)	47.1	20.8	0.034
Diabetes mellitus (%)	17.6	13.2	0.649
Pancreatic disease (%)	47.1	37.7	0.495
Cardiac disease (%)	0	3.8	0.416
Oxygen therapy (%)	5.9	3.8	0.709
ESS score	5.65 ± 3.93	3.87 ± 3.35	0.072
EDS (%)	11.8	5.7	0.395
OSA (%)	94.1	54.7	0.003
mMRC score	1.29 ± 0.47	1.32 ± 0.51	0.849
Charlson Comorbidity Index	0.29 ± 0.18	0.71 ± 0.09	0.683
Exacerbations/year	2.12 ± 1.57	1.49 ± 1.25	0.096
Hospitalizations/year	1.41 ± 0.41	0.43 ± 0.10	0.002
Disease duration (years)	17.71 ± 9.06	17.79 ± 8.21	0.971
Zung SDS score	44.24 ± 13.21	40.85 ± 10.43	0.28
Total sleep time (min)	349.41 ± 71.23	360.90 ± 68.59	0.565
Sleep efficiency (%)	78.47 ± 11.68	80.48 ± 13.26	0.579
Sleep latency (min)	44.06 ± 15.01	29.80 ± 3.54	0.178
REM latency (min)	155.29 ± 62.93	138.46	0.413
AHI	9.08 ± 5.50	7.24 ± 6.40	0.289
ODI	7.54 ± 5.93	5.83 ± 5.18	0.829
Mean oxygen saturation (%)	92.73 ± 3.45	93.29 ± 3.09	0.528
Lowest oxygen saturation (%)	86.41 ± 4.47	87.67 ± 5.71	0.407
Time with SpO_2_ < 90% (%)	15.57 ± 7.86	12.41 ± 3.84	0.699
Time with SpO_2_ < 90% (min)	33.35 ± 18.32	24.32 ± 7.81	0.722
Heart rate (bpm)	71.89 ± 9.60	67.66 ± 8.56	0.09

Abbreviations: AHI = apnea–hypopnea index; BMI = body mass index; EDS = excessive daytime sleepiness; ESS = Epworth sleepiness scale; mMRC = modified medical research council; ODI = oxygen desaturation index; OSA = obstructive sleep apnea; REM = rapid eye movements; SDS = self-related depression score.

**Table 2:** Univariate and multivariable logistic regression analyses of factors associated with anemia in the overall bronchiectasis cohort.

Variable	OR	95% CI	*p*-Value
Univariate			
Age	0.99	0.95–1.08	0.96
Female sex	9.06	1.88–43.62	0.006
Cystic fibrosis	1.6	0.53–4.83	0.405
BMI	0.88	0.76–1.04	0.134
Current/former smoker	0.35	0.04–3.04	0.342
Disease duration	0.99	0.93–1.07	0.97
mMRC score	0.89	0.29–2.73	0.847
Charlson index	0.84	0.37–1.91	0.679
Exacerbations	1.39	0.94–2.09	0.102
Hospitalization	1.99	1.21–3.23	0.006
Nutritional support	3.39	1.06–10.83	0.039
Diabetes mellitus	1.4	0.32–6.18	0.65
Pancreatic disease	1.46	0.48–4.42	0.496
Oxygen therapy	1.59	0.14–18.75	0.711
ESS score	1.14	0.98–1.32	0.08
OSA	3.42	1.64–7.21	0.015
Heart rate	1.05	0.99–1.12	0.095
Multivariate			
	Adjusted OR	95% CI	*p*-value
Age	0.98	0.93–1.05	0.66
Female sex	12.32	3.12–45.96	0.002
Hospitalization	1.1	0.49–2.42	0.813
Nutritional support	5.48	0.64–47.42	0.122
OSA	4.7	2.67–45.29	0.007

Abbreviations: BMI = body mass index; CI = confidence interval; ESS = Epworth sleepiness scale; ODI = oxygen desaturation index; OSA = obstructive sleep apnea; REM = rapid eye movements; SDS = self-related depression score; TST = total sleep time.

**Table 3:** Factors associated with anemia in patients with cystic fibrosis bronchiectasis (n = 35).

Variable	OR	95% CI	*p*-Value
Univariate			
Age	1.09	0.94–1.29	0.25
Female sex	4.33	0.76–24.61	0.098
BMI	0.91	0.70–1.17	0.452
Hospitalization	1.68	0.91–3.08	0.093
ESS score	1.16	0.95–1.40	0.131
Zung SDS score	1.06	0.99–1.13	0.094
OSA	4.24	1.88–12.25	0.045
Multivariate			
Variable	Adjusted OR	95% CI	*p*-value
Age	1.27	0.29–5.79	0.74
Female sex	1.01	0.99–1.02	0.217
OSA	1.03	0.98–1.13	0.66

Abbreviations: BMI = body mass index; CI = confidence interval; ESS = Epworth sleepiness scale; OSA = obstructive sleep apnea; SDS = self-related depression score.

**Table 4:** Factors associated with anemia in patients with non-cystic fibrosis bronchiectasis (n = 35).

Variable	OR	95% CI	*p*-Value
Univariate			
Age	0.99	0.94–1.05	0.828
Female sex	0.94	0.81–1.26	0.987
BMI	0.88	0.72–1.09	0.249
Hospitalization	2.85	1.01–8.01	0.046
Nutritional support	3.8	0.81–24.29	0.091
OSA	4.5	2.47–42.5	0.049
Multivariate			
**Variable**	**Adjusted OR**	**95% CI**	***p*-value**
Age	1.02	0.94–1.07	0.947
Hospitalization	3.69	1.06–12.84	0.04
OSA	1.12	0.96–1.31	0.136

Abbreviations: BMI = body mass index; CI = confidence interval; OSA = obstructive sleep apnea; SDS = self-related depression score.

## Data Availability

The original contributions presented in this study are included in the article. Further inquiries can be directed to the corresponding author.

## Abbreviations

The following abbreviations are used in this manuscript:

AASM	American Academy of Sleep Medicine
AHI	Apnea–Hypopnea Index
BMI	Body Mass Index
CF	Cystic Fibrosis
CI	Confidence Interval
EDS	Excessive Daytime Sleepiness
ESS	Epworth Sleepiness Scale
g/dL	grams per deciliter
IBMs	International Business Machines
IQR	Interquartile Range
mMRC	Modified Medical Research Council
NHANES	National Health and Nutrition Examination Survey
ODI	Oxygen Desaturation Index
OR	Odds Ratio
OSA	Obstructive Sleep Apnea
PSG	Polysomnography
REM	Rapid Eye Movement
SDB	Sleep-Disordered Breathing
SD	Standard Deviation
SDS	Zung Self-Rating Depression Scale
SpO_2_	Peripheral Oxygen Saturation
